# Chicago Older Adults’ Likelihood of Being Home Before and During the COVID-19 Pandemic

**DOI:** 10.1093/geroni/igab046.050

**Published:** 2021-12-17

**Authors:** Ellen Compernolle, Laura Finch, Louise Hawkley, Kathleen Cagney

**Affiliations:** 1 NORC at the University of Chicago, Chicago, Illinois, United States; 2 NORC at The University of Chicago, Chicago, Illinois, United States; 3 University of Chicago, Chicago, Illinois, United States

## Abstract

Staying at home has particularly been emphasized for older adults during the COVID-19 pandemic, given their elevated risk of infection and complications. However, little is known about the extent to which this population is indeed spending more time at home during the pandemic, compared to before it began. The present investigation addresses this question, also examining differences by gender and race/ethnicity. We analyzed ecological momentary assessments among 98 older adults (age 65-88 in 2020) who participated in two waves of the Chicago Health and Activity Space in Real Time study. Pre-pandemic data were collected from July-October 2019, and pandemic data were collected from June-September 2020. Participants responded to smartphone “pings” (five per day for 7 days in each wave; n=1,910 and n=2,437 before and during the pandemic, respectively) by reporting their momentary location (e.g., home). Findings suggest that respondents were indeed at home more often in mid-2020 than 1 year prior. Multilevel logistic regression models revealed that net of demographics, marital and employment status, and physical health, respondents were more likely to be momentarily at home during versus before the pandemic (B=0.70, SE=0.08, p<.001). This effect was larger among women than men (B=0.50, SE=0.16, p=.002), but did not differ by race/ethnicity. Additional analyses examine whether and how the observed increased reports of being at home may be associated with increased reports of momentary loneliness across the two waves. Findings characterize where Chicago older adults are spending their time amid the pandemic and how this may relate to their well-being.

